# Immune checkpoint blockade in the treatment of malignant tumor: current statue and future strategies

**DOI:** 10.1186/s12935-021-02299-8

**Published:** 2021-11-02

**Authors:** Wenwen Yang, Caining Lei, Shaoming Song, Wutang Jing, Chuanwei Jin, Shiyi Gong, Hongwei Tian, Tiankang Guo

**Affiliations:** 1 Department of Clinical Medicine, The First Clinical Medical College of Lanzhou University, Lanzhou, 730000 Gansu People’s Republic of China; 2 Department of General Surgery, Gansu Provincial Hospital, Lanzhou, 730000 Gansu People’s Republic of China; 3 The First Clinical Medicine College, Gansu University of Chinese Medicine, Lanzhou, 730000 Gansu People’s Republic of China

**Keywords:** Immunotherapy, Programmed cell death protein 1, Programmed cell death ligand-1, Immune checkpoint inhibitors, Biomarkers

## Abstract

After being stagnant for decades, there has finally been a paradigm shift in the treatment of cancer with the emergence and application of immune checkpoint inhibitors (ICIs). The most extensively utilized ICIs are targeting the pathways involving programmed death-1 (PD-1) and cytotoxic T-lymphocyte associated protein 4 (CTLA-4). PD-1, as an crucial immune inhibitory molecule, by and large reasons the immune checkpoint response of T cells, making tumor cells get away from immune surveillance. Programmed cell death ligand-1 (PD-L1) is exceptionally expressed in most cancers cells and approves non-stop activation of the PD-1 pathway in the tumor microenvironment. PD-1/PD-L1 inhibitors can block the combination of PD-1 and PD-L1, inhibit hostile to regulatory signals, and restore the activity of T cells, thereby bettering immune response. The current researchers assume that the efficacy of these drugs is related to PD-L1 expression in tumor tissue, tumor mutation burden (TMB), and other emerging biomarkers. Although malignant tumors can benefit from the immunotherapy of PD-1/PD-L1 inhibitors, formulating a customized medication model and discovering biomarkers that can predict efficacy are the new trend in the new era of malignant tumor immunotherapy. This review summarizes the mechanism of action of PD-1/PD-L1 inhibitors, their clinical outcomes on various malignant tumors, their efficacy biomarkers, as well as predictive markers of irAEs.

## Background

Immunotherapy with immune checkpoint inhibitors (ICIs) has modified the therapeutic strategy to malignant tumors and emerge as a rapidly developing area of research. It primarily changes the body’s immune system and enhances anti-tumor immunity, inhibiting and killing tumor cells. In the late nineteenth century, people first tried to use the immune system to treat cancer [1]. However, even after nearly a hundred years of research, the mechanism of the immune system to identify and fight cancer is still very controversial [2]. Today, after almost 120 years of basic research in immunology, molecular biology, virology, cell biology, and structural biology, we have further understood the role of the immune system in the regulation of tumors and the strategy of tumor cells to avoid monitoring, and subsequently decided to use immunotherapy as a promising technique to deal with the dynamic and complicated interplay between cancers and immunity [3, 4]. To defend the host from any potential threats, the immune system can do considerable damage to harmful invaders and effectively eliminate most pathological microorganisms and toxic substances. Still, the immune system must accomplish this task by weakening the checkpoint pathway of the immune response based on maintaining healthy cells and preserving its own tolerance [5].

Currently, with continuous in-depth research on the mechanism of tumor immune escape, ICIs have proven better clinical effects in the treatment of a variety of solid tumors and have become a landmark event in the history of cancer treatment. The mechanism of action of PD-1/PD-L1 in tumor immune escape and its application in tumor immunotherapy are a hot topic in current oncology research. In traditional organisms, the PD-1/PD-L1 signaling pathway plays a pivotal role in maintaining immune tolerance. In the tumorigenesis, the PD-1/PD-L1 signaling pathway can inhibit the immune response of T cells and promote the occurrence of tumor immune escape. Anti-PD-1/PD-L1 therapy has significant clinical effects, preventing the progression of advanced metastatic tumors and improving the progression the survival rate of patients to a certain extent. By the end of December 2018, the FDA-approved tumor immunotherapeutics are pembrolizumab, nivolumab, atezolizumab, avelumab, and durvalumab. Simultaneously, a range of malignant tumors has benefited from anti-PD-1/PD-L1 therapy, such as melanoma, non-small-cell lung cancer (NSCLC), small cell lung cancer (SCLC), renal cell carcinoma (RCC), classical Hodgkin lymphoma (cHL), head and neck squamous cell carcinoma (HNSCC), colorectal cancer (CRC), hepatocellular carcinoma (HCC), primary mediastinal large B-cell lymphoma (PMLBCL), Merkel cell carcinoma (MCC), etc.

However, studies [6] found that the inhibition rate of PD-1/PD-L1 inhibitors on solid tumors is solely 10–40%, which indicates that a large portion of patients can not benefit from immunotherapy. Hence, exploring accurate biomarkers for therapeutic efficacy and screening patients who benefit from PD-1/PD-L1 inhibitor therapy have become first-rate problems in the field of immunotherapy for malignant tumors. This article reviews the mechanism of action of PD-1/PD-L1 inhibitors, the application of PD-1/PD-L1 inhibitors in various malignancies and their efficacy biomarkers, as well as predictive markers of irAEs.

## Rationale of anti-PD-1/PD-L1 antibody therapy

PD-1 is a crucial immunosuppressive molecule obtained in apoptotic T-cell hybridomas. It is a type I transmembrane glycoprotein consisting of 268 amino acids with a relative molecular mass of 55,000–60,000. Its extracellular domain shares 21–33% sequence homology with CTLA-4, CD28, and ICOS [7, 8]. In the early 1990s, the American biologist Ishida et al. first discovered PD-1 when separating the transiently expressed genes involved in the programmed cell death process in apoptosis-induced mouse T cells. In typical organisms, PD-L1, on the surface of cells, can inhibit the function of lymphocytes and induce the apoptosis of activated lymphocytes after being combined with PD-1 on the surface of lymphocytes. The activation of the PD-1/PD-L1 signaling pathway can reduce immune response damage to the surrounding tissues and avoid autoimmune diseases [9]. Additionally, activation of this pathway leads to the binding of PD-L1 expressed by tumor cells to PD-1 on the surface of tumor-infiltrating lymphocytes (TILs), weakening the immune role of T cells in the local tumor microenvironment. This mediates the occurrence of tumor immune escape and promotes tumor progression [10].

**Fig. 1:**
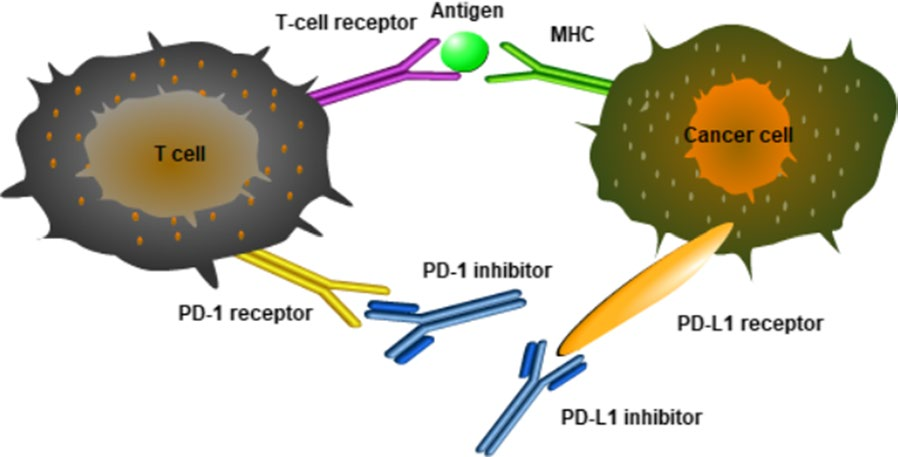
Tumor cells inhibit T cell activation by expressing PD-L1 on their cell surface, and PD-1/PD-L1 inhibitors reactivate T cells by specifically binding to PD-1/PD-L1

Studies [11] have showed that PD-L1 can be selectively and highly expressed on the surface of cancer cells, activating the PD-1/PD-L1 downstream pathway by binding specifically to PD-1 and delivering negative regulatory signals, leading to apoptosis of activated T cells and loss of immunologic activity (illustrated in Fig. 1). Therefore, the PD-1/PD-L1 pathway is a key molecule that mediates immune escape in the tumor microenvironment [12]. Targeted blockade of the PD-1/PD-L1 signaling pathway can relieve the inhibition of T lymphocytes by tumor cells, and enhance the recognition and killing effect of the immune system on foreign tumor cells. It is believed that with the deepening of research, scientists will thoroughly clarify the role and mechanism of PD-1 in the body’s immune regulation and tumor immunotherapy.

## Current clinical practice: immunotherapy approaches and checkpoint inhibition

ICIs therapy exerts antitumor effects by inhibiting the activity of immune checkpoints, releasing the “immune brake” in the tumor microenvironment and reactivating the immune response of T cells to tumors. Currently, there are two main categories of ICIs that block the PD-1/PD-L1 pathway: monoclonal antibodies against PD-1, such as nivolumab (Opdivo) and pembrolizumab (Keytruda), and monoclonal antibodies against PD-L1, such as atezolizumab (Tecentri), avelumab (Bavencio), and durvalumab (Imfinzi). Here, we review the registration trials that have successfully led to FDA approval and the commercialization of PD-1 and PD-L1 inhibitors.

## Pembrolizumab (Keytruda)

Pembrolizumab is a potent, highly selective, human-derived IgG4-kappa monoclonal antibody with potential immune checkpoint inhibitory activity and anti-tumor activity. By blocking the interaction between PD-1 and PD-L1, it helps tumors get away the immune system. It is approved for use in metastatic or unresectable melanoma, metastatic NSCLC, advanced UC, recurrent or metastatic HNSCC, cHL, HCC, CRC, malignant pleural mesothelioma (PM), MCC, MSI-H/d-MMR adult and pediatric solid tumors, advanced cervical cancer (CC), gastric or gastroesophageal junction adenocarcinoma (GEJA), and PMBCL. Table 1 lists all clinical trials of pembrolizumab for the treatment of patients with malignant tumors.

**Table 1:** Summary of pembrolizumab trials in malignant cancer

Pathology	Trial	Phase	No of. patients	Treatment	mPFS (months)	mOS (months)	ORR (%)	Other outcomes
Melanoma	KEYNOTE-006	III	834	Pembrolizumab vs. Ipilimumab	5.5 vs. 2.8	–	34 vs. 12	–
NSCLC	KEYNOTE-010	II	–	Pembrolizumab vs. Docetaxel	5.2 vs. 4.1	17.3 vs. 8.2		–
	KEYNOTE-189	III	616	Pembrolizumab + Pemetrexed and platinum vs. Placebo	8.8 vs. 4.9	–	62.9 vs. 49.4	–
HNSCC	KEYNOTE-012	Ib	60	Pembrolizumab	–	–	18	–
UC	KEYNOTE-052	II	370	Pembrolizumab	2.0	–	24	CR: 5%; DCR: 47%
	KEYNOTE-045	III	542	Pembrolizumab vs. Paclitaxel and vinflunine	–	10.3 vs. 7.4	21.1 vs. 11.4	–
cHL	KEYNOTE-087	II	210	Pembrolizumab	72.4%*	99.5%*	69	CR: 22.4%
PMBCL	KEYNOTE-170	II	49	Pembrolizumab	–	62%*	41	CR: 14%; PR: 28%
DLBCL	Frigault et al	II	29	pembrolizumab	59%*	73%*		–
MM	Badros et al	II	48	Pembrolizumab + Pomalidomide and Dexamethasone	–	–	60	CR: 8%; PR: 19%
CC	KEYNOTE-028	Ib	24	Pembrolizumab	2.0	11.0	17	DCR: 30%
	KEYNOTE-158	II	98	Pembrolizumab	2.1	9.4	13.3	DCR: 30.6%
Ovarian cancer	KEYNOTE-028	II	26	Pembrolizumab	1.9	13.8	11.5	SD: 26.9%
Endometrial cancer	Makker et al	II	23	Pembrolizumab + lenvatinib	–	–	39.6	DCR: 86.8%
TNBC	KEYNOTE-086	II	–	Pembrolizumab	2.0	9.0	5.3	DCR: 7.6%
MSI-H and dMMR^S^	KEYNOTE-164	II	63	Pembrolizumab	–	–	26.2	–
PM	Cedrés et al	Ib	25	Pembrolizumab	–	–	28	SD: 48%; DCR: 76%
GEJA	KEYNOTE-059	II	143	Pembrolizumab	–	–	13.3	DOR: 16.3 months
CRC	Ribas et al	Ib	–	Pembrolizumab + Oncolytic virus	–	–	62	CR: 33%
HCC	KEYNOTE-224	II	104	Pembrolizumab	–	12.9	17	CR: 1%; PR: 16%; DCR: 60%

NSCLC: non-small cell lung cancer; HNSCC: head and neck squamous cell carcinoma; UC: urothelium carcinoma; cHL: classic Hodgkin lymphoma; PMBCL: primary mediastinal large B-cell lymphoma; DLBCL: diffuse large B cell lymphoma; MM: multiple myeloma; CC: cervical cancer; TNBC: triple-negative breast cancer; MSI-H and dMMRS: unresectable or metastatic solid tumors, including colorectal, endometrial and other gastrointestinal cancers; PM: pleural mesothelioma; GEJA: gastric or gastroesophageal junction adenocarcinoma; CRC: colorectal cancer; HCC: hepatocellular carcinoma; mPFS: median progression-free survival; mOS: median overall survival; ORR: objective response rate; CRR: complete response; PRR: partial remission; DCR: disease control rate; DOR: duration of response; SD: stable disease; *: PFS or OS rate. –: Not available

In the current setting, data from a single-center, open-label, phase III trial [13, 14] (KEYNOTE-006) of pembrolizumab or ipilimumab monotherapy in the treatment of advanced or unresectable melanoma was recently published. The primary endpoint of median progression-free survival (mPFS) and objective response rate (ORR) was met and favored of the pembrolizumab group: 5.5 vs. 2.8 months, 34% vs. 12%. The benefit of combination ICI with chemotherapy in the first-line setting of NSCLC is well established and has been studied in phase III trials [15] (KEYNOTE-189). KEYNOTE-189 investigated the addition of pembrolizumab to pemetrexed and platinum as first-line treatment of NSCLC. Eligible patients received chemotherapy plus either pembrolizumab or placebo, followed by maintenance. The addition of pembrolizumab improved mPFS and ORR: 8.8 vs. 4.9 months, 69.2% vs. 49.4%. A phase Ib, placebo-controlled, randomized KEYNOTE-012 [16], tested the safety and efficacy of pembrolizumab treatment with any level of PD-L1 expression (at least 1% of tumor cells or stroma positive by immunohistochemistry for PD-L1) in the HNSCC. The results showed an acceptable safety profile for the drug, with an ORR of 18% for all patients and 25% vs. 14% for HPV-positive and negative patients. This suggests that patients can benefit regardless of HPV infection status. The efficacy of combining pembrolizumab with or without paclitaxel and vincristine with chemotherapy was investigated in the phase II trial KEYNOTE-052 [17] and phase III KEYNOTE-045 [18] in the treatment of UC. During the phase II trial KEYNOTE-052, the mPFS and ORR in the total population were 2.0 months and 29%. During the phase II trial KEYNOTE-052, the total mPFS and ORR were 2.0 months and 29%. The disease control rate (DCR) was 47% among the responding patients and the complete response (CR) rate was 5%. Additionally, in the phase III KEYNOTE-045, the median overall survival (mOS) was 10.3 months in the pembrolizumab group and 7.4 months in the chemotherapy group, with ORRs of 21.1% and 11.4% in the two arms, respectively. Based on these results, FDA’s accelerated approval was granted.

Information from a single-arm, open-label, phase II trial (KEYNOTE-087) [19] of pembrolizumab monotherapy for cHL was recently published. This trial was divided into three cohort groups: cohort 1 was formed by cHL patients who progressed after autologous stem cell transplantation (ASCT) and subsequent medication of brentuximab vedotin (BV) treatment, cohort 2 consisted of cHL patients who progressed after chemotherapy and BV treatment, and cohort 3 was cHL patients who had received ASCT but without BV. All adult patients received pembrolizumab. The total ORR was 69% (73.9%, 64.2% and 70.0%, respectively). The PFS and OS rates were 72.4% and 99.5%. Moreover, the benefit of combination pembrolizumab with chemotherapy in the first-line setting of non-Hodgkin lymphoma (NHL) is well established and has been studied in three phase II trials. In study KEYNOTE-170 [20], PMBCL patients were treated with pembrolizumab monotherapy and the OS rate had been met and was in favor of the pembrolizumab group (62%). Notedly, pembrolizumab delivered a high response rate with an ORR of 41%, including 14% CR and 28% PR. Frigault et al. (NCT02362997) [21] evaluated the efficacy and safety of consolidation remedy with pembrolizumab in sufferers with relapsed or refractory DLBCL. The results in the 29 patients indicated that the PFS and OS had been met (59% and 73%). Similarly, Badros et al. also assessed the phase II trial (NCT02289222) [22], where 48 patients with relapsed or refractory MM were treated with pembrolizumab combination with pomalidomide and dexamethasone. The primary endpoints of ORR validated statistical significance in favor of the pembrolizumab plus chemotherapy cohort (60%) with CR in 8% and PR in 19% of the patients. These results prompted the solely approval of pembrolizumab for hematological malignancy.

Furthermore, the safety and effectiveness of pembrolizumab in gynecologic oncology have also been demonstrated. The efficacy of pembrolizumab alone for first-line treatment of CC was presented from the randomized phase II KEYNOTE-158 trial [23] and phase Ib KEYNOTE-028 trial [24]. In phase II KEYNOTE-158 trial, pembrolizumab was approved for the treatment of patients with relapsed or metastatic CC whose disease progressed during or after chemotherapy, with 83% of patients positive for PD-L1 expression. Similarly, significant differences in mPFS, mOS, ORR, and DCR were observed (2.1 months, 9.4 months, 13.3%, and 30.6%, respectively). In the phase Ib KEYNOTE-028 trial, a total of 24 patients were treated with pembrolizumab: mPFS was 2.0 months, mOS was 11.0 months, ORR reached 17%, and DCR was 30%. A phase II KEYNOTE-028 trial [25] demonstrated pembrolizumab improved both mPFS (1.9 months) and mOS (13.8 months) in the therapy of ovarian cancer. Simultaneously, 26.9% of patients reached stable disease (SD) and the ORR reached 11.5%. Makker et al. (NCT02501096) [26] explored the efficacy of pembrolizumab in aggregate with lenvatinib in advanced endometrial cancer. The principal endpoint of ORR and DCR had been met: 39.6% and 86.8%. Besides, the phase II KEYNOTE-086 trial [27] confirmed that pembrolizumab could benefit TNBC patients more without other treatment, with an ORR of 5.3%, DCR of 7.6%, mPFS of 2 months, and mOS of 9 months, respectively.

In May 2017, FDA authorized the use of pembrolizumab for patients with unresectable or metastatic solid tumors bearing either of these two biomarkers referred to as microsatellite instability-high (MSI-H) or mismatch repair deficient (d-MMR) based on the phase II KEYNOTE-158 trial (https://clinicaltrials.gov/ct2/show/NCT02628067). The ORR was reported as 42.9%. In June 2015, pembrolizumab was accredited to treat patients with malignant PM based on the phase Ib trial [28]. The ORR reached 28%, with 48% of SD and 76% of DCR. A phase II KEYNOTE-059 trial [29] available to date in patients with advanced GEJA indicates the positive effects of pembrolizumab. The ORR reached 13.3% and DOR was 16.3 months. As of now, a single-arm, phase Ib trial [30] demonstrated the safety of presurgical and postsurgical pembrolizumab plus oncolytic virus in a set of advanced CRC patients. The ORR reached 62%, including 33% CR. Also, an increase in CD8^+^ T cell density and upregulation of PD-L1 expression was observed after treatment, suggesting that lysing viruses can improve the tumor microenvironment and have a synergistic effect with ICIs. At last, in November 2018, the FDA approved pembrolizumab for patients with sorafenib or sorafenib-intolerant HCC based on the single-arm multicenter phase II KEYNOTE-224 trial [31]. The results confirmed an ORR of 17%, DCR of 60%, mOS of 12.9 months, CR rate of approximately 1%, and PR rate of 16%.

## Nivolumab (Opdivo)

The humanized monoclonal IgG4 anti-PD-1 antibody nivolumab is approved for the treatment of metastatic melanoma, metastatic NSCLC, UC, advanced RCC, HNSCC, cHL, CRC, ovarian cancer, FL (follicular lymphoma), AML (acute myeloid leukemia), CLL (chronic lymphocytic leukemia), DLBCL, and HCC. Nivolumab works by binding to the PD-1 receptor and blocking its interaction with PD-L1 and PD-L2, thereby releasing the PD-1 pathway-mediated immunosuppressive effects on tumor cells. Completed trials using nivolumab to target malignant tumors are listed in Table 2.

**Table 2:** Summary of nivolumab trials in malignant cancer

Pathology	Trial	Phase	No of. patients	Treatment	mPFS (months)	mOS (months)	ORR (%)	Other outcomes
Melanoma	CheckMate-037	III	370	Nivolumab vs. ICC	–	–	31.7 vs. 10.6	–
	CheckMate-067	III	945	Nivolumab + ipilimumab vs. Monotherapy (Ipilimumab or Nivolumab)	11.5 vs. 6.9 vs. 2.9	–	50 vs. 40 vs. 14	–
NSCLC	CheckMate-017	III	272	Nivolumab vs. Docetaxel	–	9.2 vs. 6.0		–
HNSCC	CheckMate-141	III	361	Nivolumab vs. ICC	–	7.5 vs. 5.1	13.3 vs. 5.8	–
UC	CheckMate-275	II	270	Nivolumab	2.0	–	19.6	–
cHL	CheckMate-205	II	243	Nivolumab	14.7	–	69	CR: 16%; DOR: 16.6 months
FL	Lesokhin et al	Ib	–	Nivolumab	–	–	40	–
DLBCL	Cao et al	–	11	Nivolumab + Anti-CD19 CAR-T cell therapy	–	–	81.81	CR: 45.45%
AML	Daver et al	II	70	Nivolumab + Azacitidine vs. Demethylation medication	–	6.3 vs. 4.6	33 vs. 20	CR: 8%; PR: 19%
CLL	Jain et al	II	–	Nivolumab + Ibrutinib	–	–	43	–
Ovarian cancer	Hamanishi et al	II	20	Nivolumab	3.5	20.0	15	DCR: 45%
RCC	CheckMate-214	III	847	Nivolumab + Ipilimumab vs. Sunitinib	11.6 vs. 8.4	75%* vs. 60%*	42 vs. 27	CR: 9% vs. 1%

NSCLC: non-small cell lung cancer; HNSCC: head and neck squamous cell carcinoma; UC: urothelium carcinoma; cHL: classic Hodgkin lymphoma; FL: follicular lymphoma; DLBCL: diffuse large B cell lymphoma; AML: acute myeloid leukemia; CLL: chronic lymphocytic leukemia; RCC: renal cell carcinoma; HCC: hepatocellular carcinoma; mPFS: median progression-free survival; mOS: median overall survival; ORR: objective response rate; CR: complete response; PR: partial remission; DCR: disease control rate; DOR: duration of response; ICC: Investigator's choice of chemotherapy; *: PFS or OS rate. –: Not available

In the adjuvant setting, data from a multi-Center, open-label, phase III trial (CheckMate-037) [11] of nivolumab or investigator’s choice of chemotherapy in the treatment of advanced or unresectable melanoma was recently published. The primary endpoint of ORR had been met and favored the pembrolizumab group: 31.7% vs. 10.6%. Notably, the efficacy of combining nivolumab with ipilimumab or monotherapy was investigated in the phase III trial CheckMate-067 [32] which included 945 patients in total. The primary endpoint of mPFS and ORR was met and favored of combining nivolumab with ipilimumab group: 11.5 vs. 6.9 vs. 2.9 months, 50% vs. 40% vs. 14%. In March 2015, nivolumab was approved for the treatment of NSCLC based on the phase III CheckMate-017 trial [33], whose patients were randomly assigned to the nivolumab arm and the docetaxel arm. The primary endpoint of mOS was safety and favor of the nivolumab group: 9.2 vs. 6.0 months. In November 2016, nivolumab became the first FDA-approved immunotherapy for the treatment of relapsed or metastatic HNSCC based on phase III randomized trial (CheckMate-141) [34]. The nivolumab group had a 30% lower risk of death than the control group (investigator’s choice of chemotherapy). The mOS and ORR in the two arms had been met and nivolumab was more beneficial to patients (7.5 vs. 5.1 months, 13.3% vs. 5.8%, respectively). In February 2017, The FDA approved nivolumab for locally advanced or metastatic UC following the results from CheckMate-275 [35]. The overall ORR was 19.6% in patients with high PD-L1 expression (28.4% for PD-L1 ≥ 5%, 23.8% for PD-L1 ≥ 1%, and 16.1% for PD-L1 ˂ 1%) whereas mPFS was also higher (2.0 months).

In May 2016, nivolumab received the first approval for the treatment of patients with cHL who have relapsed or progressed after autologous hematopoietic stem cell transplantation and post-transplantation brentuximab vedotin (BV), based totally on the single-arm, phase II, multicenter trials (CheckMate-205) [36]. Nivolumab delivered a high response rate with an ORR of 69% and mPFS of 14.7 months, including 16% CR. Among responders, the DOR was maintained over time for a median of 16.6 months. Additionally, the benefit of combining nivolumab with chemotherapy in the first-line setting of non-Hodgkin lymphoma (NHL) is well established and studied in four trials. A study [37] evaluated the efficacy and safety of anti-CD19 CAR-T cell therapy in combination with nivolumab for relapsed or refractory DLBCL. The total ORR was 81.81%, including 45.45% CR. In 2016, a phase Ib trial [38] evaluated the efficacy of nivolumab in hematologic tumors, in which FL had the highest efficiency with an ORR of 40%. Also, in the phase II trial [39], patients with relapsed or refractory AML were randomized to treat with nivolumab combined with azacitidine vs. demethylation medication. The results showed that the combination therapy could benefit the patients more, where the mOS was 6.3 months vs. 4.6 months and the ORR was 33% vs. 20% in two arms, including 8% CR and 19% PR. In 2018, a phase II clinical study [40] evaluated the efficacy of nivolumab in combination with ibrutinib for relapsed or refractory CLL, with an ORR of 43%. In summary, nivolumab offers a new strategy for the treatment of hematologic malignancies.

Following platinum-based chemotherapy, a phase II trial [41] was carried out with advanced or metastatic ovarian cancer patients. The ORR of 15% was demonstrated, including 45% DCR. The mPFS was 3.5 months, and the mOS was 20.0 months. CheckMate-214 [42] investigated the addition of nivolumab to ipilimumab as the first-line treatment of RCC. Eligible patients received nivolumab plus ipilimumab vs. sunitinib monotherapy, followed by maintenance. The results proved that the addition of nivolumab improved PFS (11.6 months vs. 8.4 months), a statistically significant difference in OS rate was narrowly missed (75% vs. 60%). Nivolumab arms delivered a high response rate with ORR (42% vs. 27%), including higher CR (9% vs. 1%).

## Atezolizumab (Tecentriq)

Atezolizumab is a human anti-PD-L1 IgG1 monoclonal antibody that activates T cells and the adaptive immune system primarily by inhibiting the action of PD-L1, which in turn induces antibody-dependent cell-mediated cytotoxicity. It has been approved to treat melanoma, metastatic NSCLC, UC, CRC, and other diseases. Completed trials using atezolizumab to target malignant tumors are listed in Table 3.

**Table 3:** Approved therapies based on PD-1/PD-L1 blockade

Pathology	Trial	Phase	No of. patients	Treatment	mPFS (months)	mOS (months)	ORR (%)	Other outcomes
Atezolizumab								
Melanoma	IMspire150	III	–	Atezolizumab + Cobitinib + Verofini vs. Placebo + Cobitinib + Verofini	15.1 vs. 10.6	–	–	DOR: 21.0 vs. 12.6 months
NSCLC	POPLAR	II	287	Atezolizumab vs. Docetaxel	–	12.6 vs. 9.7	15	–
	OAK	III	1125	Atezolizumab vs. Docetaxel	–	13.8 vs. 9.6		–
UC	IMvigor210	II	310	Atezolizumab	2.1	7.9	15	–
	IMvigor211	III	931	Atezolizumab vs. ICC	–	11.1 vs. 10.6	23 vs. 22	DOR: 15.9 vs. 8.3 months
CRC	Bendell et al	I	23	Atezolizumab + Cobitinib	–	72%*		PR: 17%
Avelumab								
MCC	JAVELIN	II	88	Avelumab	–	–	31.8	CR: 9.1%; PR: 22.7%
UC	Patel et al	Ib	–	Avelumab	1.5	7.4	17	CR: 6%; PR: 11%
Ovarian cancer	Disis et al	I	–	Avelumab	–	–	9.7	DCR: 54%
Durvalumab								
NSCLC	PACIFIC	III	713	Durvalumab vs. Placebo	16.8 vs. 5.6	–	–	DOR: 72.8% vs. 46.8%
UC	Powles et al	I/II	191	Durvalumab	1.5	18.2	17.8	CR: 4.7%
Ovarian cancer	MEDIOLOA	II	32	Durvalumab + Olaparib	–	–	63	–

NSCLC: non-small cell lung cancer; UC: urothelium carcinoma; CRC: colorectal cancer; MCC: merkle cell carcinoma; mPFS: median progression-free survival; mOS: median overall survival; ORR: objective response rate; PR: partial remission; CR: complete remission; DOR: duration of response; DCR: disease control rate; ICC: Investigator's choice of chemotherapy; *: PFS or OS rate. –: Not available

In July 2020, the FDA approved atezolizumab or placebo in combination with cobimetinib and verofinil for the treatment of patients with advanced melanoma based on the Phase III IMspire150 trial [43]. The two primary endpoints of the trial, PFS and DOR, demonstrated statistical significance in favor of the atezolizumab plus chemotherapy cohort: PFS 15.1 months vs. 10.6 months and DOR 21.0 months vs. 12.6 months. In October 2016, based on the results of the phase II POPLAR [44] and phase III OAK [45] trials, atezolizumab was approved for the treatment of metastatic NSCLC with disease progression during or after platinum-containing chemotherapy. In the phase II POPLAR trial, an overall ORR of 15% was achieved, with an ORR of 26% for PD-L1 ≥ 5% and 18% for PD-L1 ≥ 1%. At a minimum follow-up of 13 months, atezolizumab significantly improved OS compared with Docetaxel (12.6 months vs. 9.7 months). In the OAK trial, OS was significantly improved in the atezolizumab arm compared with the polygalactin arm (13.8 months vs. 9.6 months). Atezolizumab also improved OS in the subgroup of patients with low or undetectable PD-L1 expression (12.6 months vs. 8.9 months). In brief, atezoluzimab achieved a significant OS benefit, with greater advantage in patients with higher PD-L1 expression in two trials.

In May 2016, an open, multicenter, single-arm phase II IMvigor210 trial (n = 310) evaluated the clinical efficacy of atezolizumab in patients with platinum-resistant locally advanced or metastatic UC. Rosenberg et al. [46] reported data from cohort 2 with an ORR of 15%, mOS of 7.9 months, and mPFS of 2.1 months. Also, on this basis, the phase III IMvigor211 trial (NCT02302807) [47] (n = 931) reported the clinical efficacy and safety of atezolizumab compared with ICC in UC patients. Results showed no significant improvement in mOS with atezolizumab compared to the chemotherapy arm (11.1 months vs. 10.6 months), and ORR was similar (23% vs. 22%). Still, DOR was longer with atezolizumab than with chemotherapy (15.9 months vs. 8.3 months). Bendell et al. [48] reported the results of a study of the atezolizumab combined with cobitinib in patients with CRC, in which 17% of patients achieved PR and the OS rate was 72%. It is unclear whether these results differ from those of chemotherapy alone.

## Avelumab (Bavencio)

Avelumab is also an IgG1 antibody directed against PD-L1, which was approved in 2017 for the treatment of patients with metastatic MCC, ovarian cancer, or metastatic UC etc. (Table 3).

In March 2017, avelumab was approved to treat patients with metastatic MCC based on the phase II JAVELIN trial [49]. In this multicenter, international, prospective, single-arm, open-label phase II trial, an ORR of 31.8% was achieved in patients with stage IV chemotherapy-refractory, histologically confirmed MCC, including 9.1% CR and 22.7% PR of the patients. As a result, the accelerated approval of Avelumab allows it to address unmet medical needs using clinical trial data that are believed to predict clinical benefit for patients. In an open, multicenter, single-arm phase Ib trial (NCT01772004) [50], avelumab demonstrated clinical efficacy and safety in patients with platinum-refractory UC. The results indicated that the PFS and OS had been met (1.5 months and 7.4 months). The ORR of 17% was demonstrated with CR in 6% and PR in 11% of the patients. A phase I trial [51] was carried out with advanced or metastatic ovarian cancer patients, following platinum-based chemotherapy. An ORR of 9.7% was demonstrated with DCR in 54% of the patients.

## Durvalumab (Imfinzi)

Durvalumab is a monoclonal IgG1k antibody approved only by the FDA in 2017 for the treatment of NSCLC, UC, and ovarian cancer (Table 3).

In February 2018, durvalumab was approved for the treatment of patients with stage III NSCLC based on the phase III PACIFIC trial [52]. The primary endpoint of mPFS and DOR had been met and favored the durvalumab arm: 16.8 vs. 5.6 months, 72.8% vs. 46.8%. Lately, Powles et al. [53] have confirmed the effectiveness of durvalumab in UC patients: ORR of 17.8% in 191 patients and CR of 4.7%. Besides, the primary endpoint of PFS and OS had been met and was in favor of the durvalumab arm: 1.5 months and 18.2 months. In 2018, durvalumab was used in combination with olaparib for the treatment of platinum-sensitive ovarian cancer patients in an open-label, single-arm, phase II study (MEDIOLOA) [54]. The results of the study showed an ORR of 63% in 32 patients treated with the combination. The combination therapy has fewer adverse reactions and no dose-limiting toxicity has been reported.

## Toxicities of PD-1/PD-L1 signal blocking

Although ICIs enhance the antitumor t-cell response, they may still lead to the development of various immune-related adverse events (irAEs) that are usually considered well-tolerated and manageable, such as interstitial pneumonia, colitis with gastrointestinal perforation, immune platelets after steroid therapy reduction, neutropenia, and sepsis [55]. A recent meta-analysis evaluated the safety and tolerability of PD-1/PD-L1 inhibitors in 3450 patients with advanced cancer from seven randomized controlled studies [56]. Compared with chemotherapy, PD-1/PD-L1 inhibitors significantly reduced the risk of fatigue, diarrhea, anorexia, nausea, and constipation. Among them, the proportions of grade 1–4 AEs, ≥ 3 AEs and discontinuation of treatment were 67.6% vs. 82.9%, 11.4% vs. 35.7% and 4.5% vs. 11.1%, respectively. While most irAEs are manageable and can be managed by clinicians, some can seriously endanger patients' lives, such as cardiac arrest, heart failure, and myocardial infarction.

In July 2017, the FDA has placed an emergency hold on several clinical trials of nivolumab, pembrolizumab, and durvalumab-containing regimens for various hematologic malignancies based on safety concerns from KEYNOTE-183 [57]. Therefore, early prevention, early recognition of grade 1 and 2 adverse reactions, and timely intervention are critical and can significantly reduce patient morbidity and mortality [58]. Notably, the use of drugs such as steroids to manage side effects does not seem to affect the efficacy of immune checkpoint inhibitors [59], which opens new paths for future research on how to reduce adverse drug reactions. Furthermore, optimization of relevant guidelines and specific care approaches facilitate early intervention and management of irAEs.

In a nutshell, the clinical manifestations of irAEs are complex and affect the quality of life of patients, and severe irAEs even require discontinuation of ICIs, which delays patient treatment. Therefore, it is particularly important to find biomarkers that predict irAEs in tumor patients treated with ICIs.

## Predictive biomarkers to assess the efficacy of ICIs

### Tumor mutation burden (TMB)

Tumor mutation burden (TMB) is defined as the total number of base substitutions (including synonymous mutations) per megabase in the coding region of the target gene. In short, it is the total number of somatic mutations in the tumor genome after removing germline mutations. The CheckMate 026 study demonstrated significantly improved PFS and OS in patients with high TMB (≥ 243 missense mutations) treated with nivolumab compared with conventional chemotherapeutic agents [60]. The POPLAR study analyzed the relationship between blood TMB (bTMB) and clinical benefit. Among patients with bTMB ≥ 10, ≥ 16, and ≥ 20, atezolizumab treated patients had an increased benefit in PFS and OS, with the greatest benefit in patients with bTMB ≥ 16 [61]. In addition, the results of the CheckMate 227 study [62] showed that nivolumab plus low-dose ipilimumab treatment significantly prolonged patients’ 1-year PFS (7.2 months vs. 5.4 months) compared with platinum-based doublet chemotherapy in patients with advanced NSCLC with TMB ≥ 10. Although TMB is considered to be a good predictor of immunotherapy, there are limitations in clinical practice. Immune nonresponse occurs in patients with high TMB, while patients with low TMB produce good immune effects. In the future, we need many prospective trials to investigate how TMB can be effectively combined with PD-L1 expression levels to predict the efficacy of ICIs jointly. Furthermore, how HLA genotypes and other germline variants affect the effect of TMB and the response to ICIs needs to be further explored.

## PD-L1 expression status

Currently, the detection of PD-L1 mainly relies on immunohistochemistry (IHC) in clinical practice. The results of KEYNOTE 024 [63] showed that patients with advanced NSCLC with high PD-L1 expression (≥ 50%) had better OS, PFS with pembrolizumab compared to conventional chemotherapeutic agents. Nevertheless, the efficacy of immunotherapy was comparable to that of conventional drug chemotherapy when PD-L1 expression was ˂ 50%. Therefore, the higher the expression level of PD-L1, the better the immunotherapy effect of NSCLC. CheckMate 012 study [64] illustrated that nivolumab in combination with ipilimumab in advanced NSCLC was more than 90% effective in patients with PD-L1 ≥ 50%. Nevertheless, CheckMate 017 and OAK trials manifested that the expression level of PD-L1 in tumor cells might not be an appropriate biomarker to predict the effectiveness of immunotherapy [33, 45]. This may be because specific signaling pathways promote malignant behavior of cancer cells, such as EGFR, mitogen-activated protein kinase (MAPK) and phosphatidylinositol 3-kinase protein kinase (PI3K AKT). The current PD-L1 detection platforms are DAKO and Ventana, and it is recommended to detect antibodies 22C3 and 28-8 using the DAKO detection platform and SP142 and SP263 using the Ventana detection platform. Whereas, due to the different antibodies used by different companies’ testing platforms, the different thresholds set for positivity, and the subjective nature of the interpretation, this leaves a great deal of uncertainty in the test results [65]. In brief, concerning patients with low or absent PD-L1 expression capacity, or patients with high PD-L1 expression capacity who urgently need cell induction, we prefer combination regimens containing ICIs and histologically selected platinum double chemotherapy to achieve higher clinical benefit.

## Tumor-infiltrating lymphocytes (TILs)

Tumor-infiltrating lymphocytes (TILs) are mononuclear immune cells distributed in tumor cell clusters and mesenchyme and are usually stained with hematoxylin–eosin (HE) and evaluated semi-quantitatively under light microscopy [66]. Since the action of PD-1/PD-L1 inhibitors requires the involvement of lymphocytes in the vicinity of the tumor, the degree of TILs infiltration can also be used as a biomarker to predict the efficacy of PD-1/PD-L1 inhibitors. In cancer tissues, TILs consist mainly of CD8^+^ and CD4^+^ T cells, followed by regulatory T cells and B cells [67]. Infiltration of TILs, especially CD8^+^ T cells, often indicates good immunotherapy response and prognosis [68]. In TNBC patients, high infiltration of CD8^+^ TILs is associated with high response rates to immune checkpoint inhibitors [69]. Previous studies have shown [70] that patients with metastatic melanoma infiltrated with high CD8^+^ TILs in the tumor tissue and tumor margins respond more significantly to immunotherapy compared to conventional cytotoxic chemotherapy. It was found [71] that NSCLC patients effectively treated with pembrolizumab had a much higher number of CD8^+^ and cut edge infiltrates in baseline biopsy specimens than patients with progressive disease. It is believed that as the study progresses, the comprehensive prediction model combining TILs with PD-L1 or TMB expression status will eventually become the most accurate biomarker for tumor immunotherapy.

## Tumor microenvironment (TME)

Many cytokines and tumor-derived exosomes in the tumor microenvironment (TME) can induce PD-L1 expression and promote tumor immune escape. TME is mainly composed of the vascular system, extracellular matrix (ECM), other non-malignant cells surrounding the tumor, and a complex network of signaling molecules that maintain the connections within the microenvironment [72]. These components promote the growth and multiplication of malignant cells, and induce their invasion and metastasis. In addition, exosomes carrying non-coding RNA are another vital component of the tumor microenvironment, contributing to tumor cell growth and migration [73]. For instance, IL-12 and IL-6 can induce PD-1 upon TCR activation by altering the chromatin structure of the PD-1 gene and enhancing PD-1 transcription through activation of STAT3/STAT4. This process that requires the proximal cis-element of the PD-1 promoter as well as the transcription factors FOXO1 and NF-κB [74]. In macrophages, IFN-α can also regulate PD-1 expression by activating the JAK/STAT signaling pathway, which enhances PD-1 transcription by forming a p48/STAT1/STAT2 complex that binds to the ISRE binding site on the PD-1 promoter [75]. TNF-α upregulates PD-L1 mRNA and protein levels, mainly through activation of NF-κB and ERK1/2 signaling pathways [76]. Tumor-derived exosomes also promote the polarization of monocytes to M2 macrophages and the expression of PD-L1 in M2-polarized macrophages via STAT3 phosphorylation, further enhancing the immunosuppressive effect [77]. In conclusion, high expression of inflammatory factors in the tumor microenvironment may be an essential factor in the poor outcome of immunotherapy. The combination of anti-inflammatory drugs and PD-1/PD-L1 inhibitors may lead to better treatment outcomes for cancer patients.

## Gut microbiome

The diversity and composition of the intestinal flora have recently been found to significantly influence the efficacy of treatment with ICIs in oncology patients [78]. It has been shown that the efficacy of treatment with ICIs decreases with the use of antibiotics and is better in certain specific intestinal microorganisms, such as *Bifidobacterium* spp., *Eckermannia* spp. and *Bacteroidetes* spp. Based on the results of a retrospective multivariate analysis, receipt of antibiotics before immunotherapy was a negative predictor of long-term patient survival. Routy et al. [79] found that the relative abundance of *Ekmania* spp. was significantly associated with good clinical outcomes in patients with advanced NSCLC and UC. Immunoassays showed higher densities of melanoma-infiltrating CD8^+^ T cells and higher frequencies of circulating CD8^+^ and CD4^+^ T cells in responding patients with a good gut microbiome [80]. All these findings suggest that the gut microbiome of oncology patients can significantly influence the efficacy of anti-PD-1 therapy. It has been reported that if antibiotics are used within 30 days prior to the start of treatment with ICIs, then patients have significantly lower PFS and OS [81]. Therefore, in future studies, perhaps we can use phages as highly selective tools to specifically eliminate negative bacteria as potential intervention tools to enhance the efficacy of immunotherapy.

## Microsatellite instability-high (MSI-H) or mismatch repair deficient (d-MMR) status

Mismatch repair deficient (d-MMR) is an important mechanism for avoiding gene replication errors, which prevents mutations and repairs polymerase errors during replication and is essential for genetic gene stability. Several clinical studies have shown that MSI-H/dMMR patients are more likely to benefit from immunotherapy [82]. In addition, the FDA has approved pembrolizumab for the treatment of MSI-H/dMMR-expressing positive solid tumors, which presented that 39.6% of patients achieved complete or partial remission and 78% of patients had a duration of drug response of 6 months or longer [83]. CheckMate142 demonstrated that compared to nivolumab monotherapy, the combination of nivolumab and pembrolizumab in patients with MSI-H/dMMR metastases was more effective than nivolumab [84]. A recent clinical trial supports this view even more [85]. The study pointed out that the pembrolizumab group had a significantly higher PFS and OS (16.5 months, 43.8%) than the chemotherapy group (8.2 months, 33.1%), at a median follow-up time of 32.34 months.

## Lymphocyte and monocyte ratio (LMR)

In 2020, a retrospective analysis investigated the predictive value of LMR in patients with advanced tumors using PD-1 inhibitors to identify patients who might have a better response to PD-1 inhibitors [86]. The optimal cut-off values for LMR were delineated using the working characteristic curves of the subjects, and patients were divided into high and low LMR groups. The results of the study showed that the ORR of the high and low LMR 6-week groups were 32.7% and 7.6%, respectively. LMR-6 weeks was significantly associated with the effect of anti-PD-1 antibody therapy; therefore, LMR-6 weeks can be used as an early predictor for stratification of patients with better response to anti-PD-1 drugs. Currently, studies on such trials are relatively scarce and need to be further confirmed by a large number of high-quality prospective studies.

## Human leukocyte antigen class I (HLA-I) molecules

Host genetics-related biomarkers are mainly associated with host immune gene polymorphisms, including HLA-I diversity and FcγR single nucleotide polymorphisms, which are positively correlated with the efficacy of ICIs. Correale et al. [87] reported that class I HLA allele characterization has vital implications in predicting nivolumab efficacy in mNSCLC. The study demonstrated that a poor outcome in patients negative for the expression of the two most frequent HLA-A alleles was detected (HLA: HLA-A*01 and or A*02; PFS: 7.5 vs. 15.9 months). In particular, HLA-A*01-positive patients showed a prolonged PFS of 22.6 and OS of 30.8 months, respectively. Simultaneously, several studies [88, 89] have already shown that HLA-I diversity was mainly expressed at the HLA-B and HLA-C loci: HLA-B-encoded MHCs could bind to a greater diversity TCRs, and HLA-C in APCs expression was higher than that of other cells. Moreover, HLA-I diversity could also promote the efficacy of ICIs from the perspective of TMBs and TCRs. It was shown that the increased affinity of FcγR encoded by allele CD16AV158F with IgG immunoglobulin was positively correlated with CTLA antibody efficacy in melanoma.

## Predictive biomarkers of irAEs

### Chemokines and cytokines

A study of chemokine changes in patients with irAEs found that CXCL9, CXCL10, CXCL11, and CXCL19, among the CXC subfamily of chemokines, were lower at baseline levels before treatment than in patients without irAEs. In contrast, only CXCL9 and CXCL10 showed significant increases after treatment [90]. In another study, patients with irAEs showed only an increase in chemokine CCL5 after treatment with ICIs [91]. CXCL9 and CXCL10 had functions in the tumor microenvironment, such as regulating T cell differentiation and directing the migration of immune cells to tumor tissues, while CCL5 was thought to be associated with graft-versus-host disease. In other words, these chemokines have different regulatory effects on the immune system, and monitoring their changes has a significant impact on irAEs.

### Soluble CD163

Soluble CD163 was derived from macrophages and was significantly elevated in a variety of autoimmune diseases, such as rheumatoid arthritis and common aspergillosis. CD163-positive macrophages secrete the chemokine CXCL5. Fujimura et al. [92] measured the concentrations of soluble CD163 and CXCL5 before and after 42 days of nivolumab treatment, and both CD163 and CXCL5 appeared significantly elevated in patients with irAEs compared to those without irAEs.

### HLA alleles

The development of autoimmune diseases was closely related to genetic factors, and similarly, genetic factors were associated with the development of irAEs. A study showed that the HLA-DRB1*11:01 haplotype was related to pneumonitis irAEs [93]. HLA-DRB1*03:01 was about colitis irAEs [94]. HLA-B* 52:01 and HLA-C* 12:02 were associated with arthritis irAEs [95]. HLA-B* 4002 was concerned with nivolumab for Hodgkin’s lymphoma type 1 diabetes mellitus that emerged from Bartholin’s tumor [96]. HLA-DRB1* 09:01-DQB1* 03:03 was related to nivolumab in treating of thyroiditis in renal cancer [97]. In short, accurate and real-world evidence-based multimodal definition of baseline tumor immunogenicity, as well as HLA haplotype characterization, can help identify not only patients with responders but patients at high risk for fatal irAEs [98].

## Conclusions and future prospectus

In summary, PD-1/PD-L1 inhibitors alone or in combination with other regimens are of great importance in the treatment of malignancies. And the emergence of biomarkers provides a direction for precision therapy of tumor immunity. Future prospective studies are still needed to evaluate the integration of these biomarkers with other potential factors (e.g., TILs with PD-L1 or TMB) and, in turn, to explore more precise biomarkers of efficacy for tumor immunotherapy using standardized methods and thresholds, such as liquid biopsy-based analysis. Despite the significant advances in immunotherapy, today’s studies demonstrate that most patients eventually relapse and develop severe adverse effects such that patient prognosis is compromised. Therefore, there is an urgent need to address how to find more effective biomarkers of efficacy and how to reduce the adverse effects of drugs.

In addition, targeting the innate immune system may also be an essential therapeutic tool in the future. Innate immune cells, such as macrophages, NK cells, neutrophils, and other myeloid cells, play an important role in complementing the effector activity of T cells. Various combination treatments are being investigated. In short, there is an increasing emphasis on the potential contribution of innate immune effectors against tumor immunity, and the integration of multiple means of targeting the adaptive immune system into PD-1/PD-L1 inhibitor-based therapies may be a fundamental combination approach in immunotherapy in the future. Clinical research in cancer immunotherapy is outpacing its basic research advances, creating an opportunity to combine emerging scientific and clinical insights to deepen our understanding of cancer immunity, and present a significant challenge to establish future cancer immunotherapies. It is believed that as the research progresses, immunotherapy for malignant tumors will eventually enter the era of individualized and precise treatment.

## Data Availability

Not applicable.

## Abbreviations

ICIs: Immune checkpoint inhibitors
irAEs: Immune-related adverse events
PD-1: Programmed death-1
CTLA-4: Cytotoxic T-lymphocyte associated protein 4
PD-L1: Programmed cell death ligand-1
NSCLC: Non-small-cell lung cancer
SCLC: Small cell lung cancer
RCC: Renal cell carcinoma
cHL: Classical Hodgkin lymphoma
HNSCC: Head and neck squamous cell carcinoma
CRC: Colorectal cancer
HCC: Hepatocellular carcinoma
PMBCL: Primary mediastinal large B-cell lymphoma
MCC: Merkel cell carcinoma
MSI-H/dMMR: Microsatellite instability-high or DNA mismatch repair deficient
PM: Pleural mesothelioma
CC: Cervical cancer
GEJA: Gastric or gastroesophageal junction adenocarcinoma
mPFS: Median progression-free survival
ORR: Objective response rate
DCR: Disease control rate
CR: Complete response
ASCT: Autologous stem cell transplantation
BV: Brentuximab vedotin
mOS: Median overall survival
NHL: Non-Hodgkin lymphoma
CPS: Combined positive score
SD: Stable disease
FL: Follicular lymphoma
AML: Acute myeloid leukemia
CLL: Chronic lymphocytic leukemia
TMB: Tumor mutation burden
TME: Tumor microenvironment
TILs: Tumor-infiltrating lymphocytes
LMR: Lymphocyte to monocyte ratio
HLA: Human leukocyte antigen
